# Effects of Medical Ozone Injection via Intervertebral Foramen Epidural Space on Postherpetic Neuralgia: Protocol for a Randomized Controlled and Double-Blind Clinical Trial

**DOI:** 10.2196/68847

**Published:** 2025-07-15

**Authors:** Dongmei Xiang, Lei Deng, Rui Zhou, Xianjie Zhang, Yukai Zhou, Dan Zhou, Yanhua Peng

**Affiliations:** 1 Department of Anesthesiology, Deyang People’s Hospital Deyang China; 2 Shanghai Key Laboratory of Anesthesiology and Brain Functional Modulation, Clinical Research Center for Anesthesiology and Perioperative Medicine，Translational Research Institute of Brain and Brain-Like Intelligence，Department of Anesthesiology and Periop Shanghai China

**Keywords:** herpes zoster, postherpetic neuralgia, medical ozone, computed tomography, dorsal root ganglion, pain

## Abstract

**Background:**

Previous studies have demonstrated that ozone injection into the dorsal root ganglion significantly reduces pain scores associated with herpes zoster, suggesting its therapeutic potential for managing postherpetic neuralgia (PHN) via the intervertebral foramen epidural space. However, there are no specific reports addressing the treatment of herpes zoster and PHN by involving the thoracic and lumbar nerves under computed tomography (CT) guidance. Our research focuses on the effect of medical ozone administered through the intervertebral foramen epidural space on PHN.

**Objective:**

This is a protocol for a prospective, randomized, controlled, and double-blind trial to detect whether the injection of medical ozone via the intervertebral foramen epidural space can reduce the incidence of PHN.

**Methods:**

After signing the written informed consent, patients meeting eligibility criteria will be allocated into the medical ozone group and the control group in a 1:1 ratio according to the randomized grouping information, with 35 patients in each group. Patients in both groups accepting the surgical procedure under CT guidance will be injected with 5 mL of therapeutic liquid. Subsequently, medical ozone (30 μg/mL, 5 mL in each segment) will be slowly administered in the medical ozone group versus a sham procedure in the control group. The primary outcome will be the incidence of PHN 3 months after the subsidence of rashes and vesicles. The secondary outcomes will be the times of injection treatment, complications during the surgical procedure, times of remedial analgesia with ultrasound-guided nerve block, numerical rating score and tactile sensation, Hospital Anxiety and Depression Scale score, and the use of lidocaine cataplasms and oral analgesics before and after the surgical procedure under CT guidance. Data analyses between the 2 groups will be compared using the 2-sided Student *t* test or Wilcoxon Mann-Whitney test based on the viability of the normality assumption, while chi-square or Fisher exact test will be used to compare the categorical data.

**Results:**

This study was approved by the medical ethics committee of Deyang People’s Hospital on February 27, 2024 (2024-03-002-K01). The first patient was enrolled on May 14, 2024. As of November 2024, 25 participants have been enrolled out of 70 who received screening. The analysis of the efficacy and safety data is expected to be performed in about May 2025 after all the patients have enrolled, with the approximate publication of results by September 2025.

**Conclusions:**

If this exploratory trial proves effective, medical ozone administered via the intervertebral foramen epidural space may be utilized to aid in the recovery of the infected nerves and decrease the incidence of PHN in clinical settings. Positive outcomes will bolster the potential of employing medical ozone in the treatment of herpes zoster, thereby contributing to a reduction in the incidence of PHN.

**Trial Registration:**

Chinese Clinical Trial Registry ChiCTR2400084014; https://tinyurl.com/jk6p9hn2

**International Registered Report Identifier (IRRID):**

DERR1-10.2196/68847

## Introduction

Herpes zoster (HZ) is a cutaneous disease caused by the reactivation of *Human herpesvirus 3* (varicella zoster virus) in humans [1]. The typical clinical characteristics of HZ include a cluster of rashes and vesicles on a red base, a unilateral dermatomal distribution, and intense neuralgia along the peripheral nerves near the skin lesions [2,3]. Approximately 1 out of 3 people in the United States will experience HZ within their lifetime [4]. The risk of developing postherpetic neuralgia (PHN) ranges from 5% to more than 30%, and over 30% of patients with PHN experience persistent pain for more than 1 year [5,6]. Moreover, patients with older age, immunosuppression, diabetes, systemic lupus erythematosus, rheumatoid arthritis, psychological stress, and organ transplant are more likely to experience HZ and PHN [4,6,7].

Currently, the treatment strategies for HZ include antiviral drugs, pain control, and prevention of PHN [1,7]. Studies have found that the earlier effective intervention of HZ can shorten the duration of illness, decrease the severity of HZ-related pain, and reduce the incidence of PHN [8-10]. Sufficient control of acute pain dampens the sensitization of peripheral nociceptors and the hyperexcitability of the central nervous system [11,12]. Although there are many methods to control HZ-related pain, including nerve block [13], pulsed radio frequency treatment [11,14], computed tomography (CT)-guided steroid injection [7], continuous epidural analgesia [15], and use of HZ vaccine and drugs [16,17], intractable PHN still remains [18]. More than 5% of the older patients experience PHN for more than 1 year after an acute herpes infection [19].

Ozone, consisting of triatomic oxygen atoms, is a highly reactive oxidant molecule, which is widely used in the clinic for pain management and several disease treatments [20,21]. Ozone can stimulate the body-repairing system, show anti-inflammatory effects, activate inhibitory interneurons, and generate analgesic effects via the release of enkephalin and endorphin, which also directly act on the inflammatory tissues around the nerves to alleviate pain [20]. Medical ozone therapy is also helpful for the treatment of zoster-associated acute pain [7]. Although the exact mechanism underlying ozone-related analgesia is still not understood completely, several studies have indicated that ozone therapy is able to treat PHN without tissue damage and complications [7,22,23]. Ozone autohemotherapy combined with pharmacological therapy has been reported to be superior to isolated pharmacological therapy in patients with PHN and is an effective and safe way to relieve PHN [23]. In a previous research, therapy under CT guidance combined with ozone injection–treated C3-C8 HZ neuralgia was safe and effective, and the numerical rating score (NRS) decreased significantly [7]. Ozone reaches the site of the involved intervertebral foramen epidural space segment under CT guidance, directly acting on the affected dorsal root ganglion (DRG) infected by the varicella zoster virus, where the virus establishes lifelong latency, and DRG is an essential site of generation of pain in HZ. Furthermore, DRGs lie at the entrance to the spinal cord receiving input from peripheral nerve terminals. Early effective treatment of HZ is beneficial for the prevention of PHN. However, the use of medical ozone injection–treated thoracic and lumbar nerves via the intervertebral foramen epidural space under CT guidance on PHN has not yet been investigated. Therefore, we speculate that medical ozone injected via the intervertebral foramen epidural space may reduce the incidence of PHN.

Here, we present the protocol for a prospective, randomized, controlled, and double-blind trial to evaluate whether the injection of medical ozone via the intervertebral foramen epidural space can show therapeutic effects for the infected nerve and reduce the incidence of PHN.

## Methods

### Study Design

**Figure 1:**
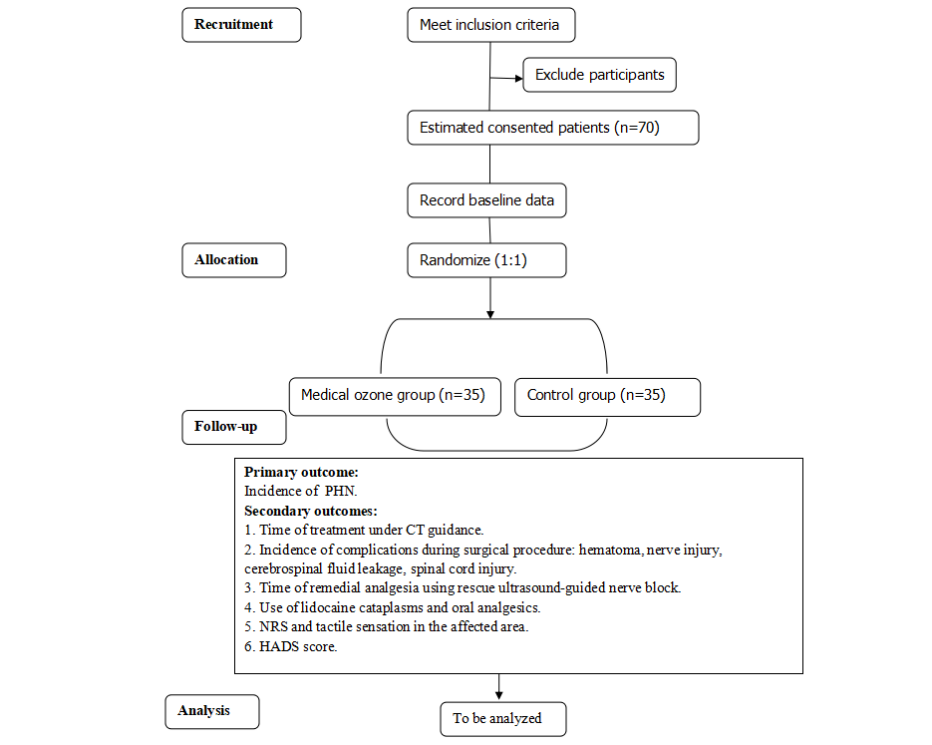
The flowchart for this study. CT: computed tomography; HADS: Hospital Anxiety and Depression Scale; NRS: numerical rating scale; PHN: postherpetic neuralgia.

This is a prospective, single-center, double-blind, randomized controlled study to detect whether medical ozone injection via the intervertebral foramina can effectively decrease the incidence of PHN. The patients will be recruited in Deyang People’s Hospital, Deyang City, Sichuan Province, People’s Republic of China. Table 1 shows the study schedule in accordance with the SPIRIT (Standard Protocol Items: Recommendations for Interventional Trials) guidelines (Multimedia Appendix 1). Figure 1 shows the flowchart for this study. All researchers will be trained to conduct the study by using a standard and uniform protocol.

**Table 1:** The study schedule of the enrollment, interventions, and assessments.

			Study period															
			Enrollment		Allocation		Postallocation											
Timepoint (T)			-T1		0		T1		T2		T3		T4		T5	T6		T7
**Enrollment**																		
	Eligibility screening	✓																
	Informed consent	✓																
	Allocation			✓														
**Interventions**																		
	With medical ozone injection					✓												
	Without medical ozone injection					✓												
**Assessments**																		
	[List baseline variables]	✓		✓														
	Times of treatment					✓												
	Complications of surgery					✓												
	Times of rescue nerve block							✓		✓		✓		✓		✓	✓	
	Use of lidocaine cataplasms			✓		✓		✓		✓		✓		✓		✓	✓	
	Use of oral analgesics			✓		✓		✓		✓		✓		✓		✓	✓	
	Numerical rating score			✓		✓		✓		✓		✓		✓		✓	✓	
	Tactile sensation			✓		✓		✓		✓		✓		✓		✓	✓	
	Hospital Anxiety and Depression Scale score			✓		✓		✓		✓		✓		✓		✓	✓	

### Inclusion and Exclusion Criteria

The inclusion criteria are as follows: (1) patients who are clinically diagnosed with HZ within 90 days, (2) patients who are older than 18 years, (3) the HZ region involves the unilateral thoracic 1-12 nerves and lumbar 1-5 nerves, and (4) the patients’ NRS is higher than 4 when they come to the hospital.

The exclusion criteria are as follows: (1) patients who are unable to complete self-evaluation questionnaires; (2) patients who have contraindications to the use of medical ozone, steroids, and lidocaine, and to CT-guided puncture (puncture site infection, coagulation dysfunction, use of anticoagulants, or platelet count less than 70 × 10^9^/L); (3) patients who have a history of glucose-6-phosphate dehydrogenase deficiency, hyperthyroidism, or sickle cell anemia; (4) pregnant or lactating women; (5) patients who have hemochromatosis or copper/iron therapy; and (6) critically ill patients.

### Randomization and Blinding

Power Analysis and Sample Size (PASS 15) software (NCSS, LLC) will generate a random sequence. The allocation information is going to be concealed in opaque envelopes and maintained by a nurse who will not be involved in this study. Once a patient is recruited, according to the randomized grouping information, the patients will be allocated to the medical ozone injection group and the control group in a 1:1 ratio. This is a prospective and double-blind clinical trial where patients and observers do not know the grouping. To sustain blinding, an independent nurse will help prepare the medications in sterile syringes marked with research numbers, and the surgical operator will not participate in this study. Blindness can only be uncovered when severe adverse events occur.

Specifically, both patients and evaluators will be given questionnaires at the end of the study to investigate the grouping of patients, and the statistical results of the questionnaires will eventually be compared and analyzed with the true grouping. The blinding success rate will be evaluated to ensure that the blinding is conducted successfully.

### Surgical Procedure and Interventions

In both groups, the surgical procedure will be performed under the guidance of a CT scan. The patients will lie in a prone position on the CT imaging bed. The vital signs will be monitored, and oxygen will be provided via a nasal oxygen tube at a rate of 3 L/min during the whole procedure. Only the surgical region will be exposed to the radiation, and other body regions will be covered by lead clothing to avoid extra exposure. The puncture segments will be based on the affected skin area. A slender metal needle will be placed parallel to the spine. The accurate puncture point will be determined by the following steps: Siemens Force CT scanning will be used to locate the upper edge level of the target intervertebral foramen, and the puncture target will be selected as the dorsal point of the target intervertebral foramen level. The point of the needle will be the intersection of the CT scanning level line and the extension. Subsequently, routine skin disinfection, drape, and local anesthesia of each puncture site with 1% lidocaine will be performed. After puncturing the skin, the operator will try to place the needle on the designed pathway, moving toward the target intervertebral foramen under CT guidance. When the tip of the needle enters the spinal canal, the resistance to the needle insertion will disappear and the negative pressure test will become positive. Once more, a CT scan will be performed to determine the precise position of the puncture needle tip. If more than one segment of DRGs is affected, puncture operations at all sites will be performed simultaneously. Then, 5 mL of the therapeutic liquid will be injected into each segment, which consists of 2 mL of 2% lidocaine, 0.5 mL of compound betamethasone (betamethasone sodium phosphate 0.2 mg/mL and betamethasone dipropionate 0.5 mg/mL), 0.5 mL of vitamin B12 (50 mg/mL), up to a total volume of 5 mL with saline. Patients in the medical ozone group will receive an additional slow injection of 5 mL of medical ozone (30 μg/mL) in each segment, while a sham procedure will be administered in the control group. A CT scan will be performed once again to confirm the satisfactory spread of the liquid and gas. Then, the needle will be pulled out, and the puncture point will be covered with sterile dressing. After an additional observation for 30 minutes, the patients will be safely escorted back to the ward in a supine position.

The treatment of the patient follows the relevant principles of HZ treatment at Deyang People’s Hospital. Oral analgesics gabapentin and pregabalin can be used according to the patient’s pain level from admission to discharge. When the patient’s postoperative NRS is greater than or equal to 4 points within a week after the CT-guided treatment, rescue ultrasound-guided nerve block treatment of the affected range can be administered after excluding contraindications, and lidocaine cataplasms can be applied to the painful area of the skin after the subsidence of rashes and vesicles. After 1 week of observation and treatment after the first CT-guided treatment, if NRS is still greater than 4, the treatment under CT guidance can be repeated once, and all therapies ought to be carefully recorded in detail on case report forms on time. The treatment protocol will remain identical across both groups, with the sole distinction being the administration of medical ozone (30 μg/mL, 5 mL) in the medical ozone group versus a sham procedure in the control group, while maintaining consistency in all other therapeutic parameters, including injection technique, monitoring intervals, and adjunctive pharmacotherapy. The report of the security incidents of patients during their stay in the hospital will follow the relevant principles of Deyang People’s Hospital.

### Outcomes

Participants in both groups will be evaluated at time 0 (baseline), once after the operation procedure (time 1), 1 week (time 2), 2 weeks (time 3), 1 month (time 4), 2 months (time 5), 3 months (time 6), and 6 months (time 7) after the operative day.

#### Primary Outcomes

The primary outcome is the incidence of PHN at 3 months after the subsidence of rashes and vesicles.

#### Secondary Outcomes

The secondary outcomes are as follows.

1. Time of treatment under CT guidance.
2. Incidence of complications during the surgical procedure: the incidence of hematoma, nerve injury, cerebrospinal fluid leakage, and spinal cord injury.
3. Time of remedial analgesia using rescue ultrasound-guided nerve block.
4. Use of lidocaine cataplasms and oral analgesics at 1 week, 2 weeks, 1 month, 2 months, 3 months, and 6 months.
5. NRS and tactile sensation in the affected areas at once, 1 week, 2 weeks, 1 month, 2 months, 3 months, and 6 months after the operative day.
6. Hospital Anxiety and Depression Scale score before injection therapy under CT guidance and at once, 1 week, 2 weeks, 1 month, 2 months, 3 months, and 6 months after the operative day.

### Data Collection and Management

Baseline demographic data, including age, gender, height, body weight, comorbidity, affected nerve segments, NRS, tactile sensation in the affected area, Hospital Anxiety and Depression Scale score, use of lidocaine cataplasms, oral analgesics, and the days from the onset of pain in the affected areas to CT-guided treatment, will be recorded before the intervention by the assessors of the research team. The primary outcome and secondary outcomes will be documented in case report forms by research observers who are independent of the grouping information. All data will be inputted and stored in the SPSS software (version 26.0; SPSS Inc) by an independent researcher. LD and XZ will be responsible for managing the data. All the researchers will be trained in skills such as eligibility evaluation, randomization, privacy protection, and interview skills before enrollment.

### Data Reporting Guidelines

The SPIRIT reporting guidelines will be used for this paper.

### Sample Size

The reported incidence of PHN ranged from 5% to 30% [5]. We aimed to detect a 25% difference in the incidence of PHN between the groups. The sample size was determined by the model of compare 2 proportions, that is, the 2-sample, 2-sided equality by using the PASS 15 software. We hypothesized an α level of 0.05 and a power level of 0.8. Therefore, we estimated that 33 participants were needed in each group. Considering a 5% dropout, we decided to include 70 patients, that is, 35 in each group.

### Recruitment Plan

A recruitment advertisement will be posted on the WeChat Official Account of Deyang People’s Hospital. Print advertising will be pasted on the doors of the department of pain treatment and the dermatology department. However, no persuasive advertising will be used to help with recruitment. Researchers will introduce this study to patients with HZ. To promote participant retention and completion of follow-up, we will inform them of the time points of phone calls during the recruitment period and reserve at least 2 records of contact numbers and their home address. A follow-up visit will occur at time 2 to time 7 after the treatment under CT guidance. All participants will be asked to return to the department of pain treatment and the dermatology department to accept regular fortnightly outpatient follow-up. The recruitment is planned to begin on May 14, 2024, and end on April 30, 2025.

### Statistical Analysis

SPSS software will be used for all statistical analyses. The normality of the data distribution will be assessed using the Shapiro-Wilk test. Normally distributed data will be summarized as mean (SD), while nonnormally distributed data will be expressed as median (25th and 75th percentiles). Categorical data will be shown as frequency and percentages. Continuous data between the 2 groups will be compared using the 2-sided Student *t* test or Wilcoxon Mann-Whitney test based on the viability of the normality assumption, while chi-square or Fisher exact test will be used to compare the categorical data. Subgroup analyses will be conducted to analyze the primary and secondary outcomes based on the research findings. Sensitivity analysis for the findings will also be conducted. Significance is set as a 2-sided *P* value <.05.

### Ethical Considerations

This study was approved by the medical ethics committee of Deyang People’s Hospital on February 27, 2024 (ethics approval: 2024-03-002-K01). The ethics committee of Deyang People’s Hospital will audit the ethic-related matters of this study once a year. This study will be performed in accordance with the Declaration of Helsinki. The research team will explain this study to the eligible participants and present them with informed consent forms. After signing the informed consent forms and relevant documents, candidates will be included into the procedure. They can withdraw from the study at any time. To ensure the privacy of the patients, all personally identifiable information will be rigorously deidentified prior to data analyses. Each participant will be assigned a random number upon participating in the study as his/her ID. The study protocol (version 3.0) was registered in the Chinese Clinical Trial Registry on May 9, 2024 (registration: ChiCTR2400084014). Patients who will complete the study will receive no compensation after completion. The results of this study will be published in a peer-reviewed journal with authorship eligibility according to the criteria of the International Committee of Medical Journal Editors.

## Results

The first patient was enrolled on May 14, 2024. As of November 2024, a total of 25 participants have been enrolled out of the 70 who received screening. The analysis of the efficacy and safety data is expected to be performed in about May 2025 after all the patients have been enrolled, with an approximate publication of the results by September 2025.

## Discussion

### Anticipated Findings

The main finding of this study is the detection of the effects of medical ozone injection via the intervertebral foramen epidural space on PHN. This study aims to provide knowledge regarding the possibility of detecting medical ozone acting directly on DRGs affected by the varicella zoster virus. If the study proves that medical ozone can decrease NRS after the surgical procedure and the incidence of PHN, the results have the potential to improve the ability to prevent PHN in the future. Predicting the effects of medical ozone enables the possibility of initiating early HZ treatment, which is expected to reduce PHN and increase the quality of life among people with varicella zoster virus infection.

In the clinic, early effective treatment of HZ is beneficial for the prevention of PHN [24], because once the disease continues for more than 3 months after the onset of acute zoster, completely alleviating the pain becomes extremely difficult [25]. The transmission of peripheral pain signals to the upper signal center can be affected by the injection therapy of the epidural space, where the medicine acts directly on the spinal DRG [7,26]. This study is designed to assess whether the injection of medical ozone in combination with therapeutic liquid via the intervertebral epidural foramen space under CT guidance can reduce the incidence of PHN. PHN is very difficult to alleviate completely, despite several treatment methods reported [27]. According to Thompson et al [27], the proportion of individuals with HZ who developed PHN was higher from 2007 to 2018 than from 1994 to 2006, even though researchers and clinicians have made numerous attempts and efforts to reduce the incidence of PHN. Therefore, further research and exploration are urgently needed on the prevention and treatment of PHN.

Clinical evidence confirmed that escalating the ozone concentration from 25 μg/mL to 60 μg/mL in ozone autohemotherapy was clinically safe and effective, with no significant adverse events reported across multicenter trials [28]. A study [28] found that ozone autohemotherapy was an effective treatment for patients with acute HZ, where it rapidly and effectively alleviated pain symptoms and improved the cytokine levels [28]. However, the ozone autohemotherapy process often requires multiple sessions and may have a risk for bleeding. A previous retrospective study analyzed the clinical data of patients with zoster-associated pain treated under ultrasound-guided percutaneous ozone injection around the cervical DRG at the injured nerve level, supporting the effectiveness of percutaneous ozone injection [22]. However, that study only included 30 participants.

The combination of local anesthetics, steroids, and neurotrophic agents injected via the epidural space under CT guidance is also a common treatment method in clinical practice, usually performed once a week [7]. The early effective treatment can promote infected nervous tissue recovery, preventing the involved DRG scarring and fascial contracture in the innervated area [7]. In addition, therapy under CT guidance offers the unique capability to visualize and confirm real-time deposition of therapeutic liquid and ozone at targeted pathological sites through high-resolution anatomical imaging, thereby enhancing precision and minimizing off-target effects.

We attempt to explore whether injection of medical ozone combined with therapeutic liquid via the intervertebral foramen epidural space under CT guidance can heal the involved DRG scar and reduce the incidence of PHN since medical ozone is injected directly near the infected DRG. Thus, it is likely to help the nervous tissue recover early and completely, reducing the incidence of PHN.

### Limitations

There are some limitations in this study. First, limited by the broad and variable incidence of PHN, it is difficult to calculate a perfect sample size. Second, no strict limitations will be placed on the regimen for adjunctive therapies such as gabapentin, pregabalin, and nerve blocks, which may introduce treatment heterogeneity across participants. However, the flexibility of adjuvant therapy allowed in this study was based on the context of ethical considerations and real-world applicability, and retaining adjuvant therapy flexibility would improve extrapolation of the results and reflect ozone therapy’s incremental value in routine pain management. Based on the results of this study, we will further design multicenter and larger sample size clinical studies to detect the effects of medical ozone on PHN.

### Conclusion

If this exploratory trial proves effective, medical ozone administered via the intervertebral foramen epidural space may be utilized to aid in the recovery of the infected nerves and decrease the incidence of PHN in clinical settings. Positive outcomes would bolster the potential of employing medical ozone in the treatment of HZ, thereby contributing to a reduction in the incidence of PHN.

## Appendix

Multimedia Appendix 1 SPIRIT checklist.

## Abbreviations

CT: computed tomography
DRG: dorsal root ganglion
HZ: herpes zoster
NRS: numerical rating score
PASS: Power Analysis and Sample Size
PHN: postherpetic neuralgia
SPIRIT: Standard Protocol Items: Recommendations for Interventional Trials

## Data Availability

The datasets of this study can be obtained from the corresponding author upon reasonable request at 6 months after the publication of the study results.
